# ﻿The genus *Japonitata* Strand (Insecta, Coleoptera, Chrysomelidae, Galerucinae) in Taiwan: a redefinition of the genus and descriptions of two new species

**DOI:** 10.3897/zookeys.1125.93703

**Published:** 2022-10-26

**Authors:** Chi-Feng Lee

**Affiliations:** 1 Applied Zoology Division, Taiwan Agricultural Research Institute, Taichung 413, Taiwan Applied Zoology Division, Taiwan Agricultural Research Institute Taichung Taiwan

**Keywords:** Host plant, leaf beetles, new combination, nomenclature, *Paraplotes*, *Shairella*, taxonomy

## Abstract

The genus *Japonitata* is redefined based on comparison with its allied genera *Paraplotes* Laboissière, 1933 and *Shairella* Chûjô, 1962. *Japonitataquadricostata* Kimoto, 1996 and *J.caerulea* Kimoto, 1996 are transferred to *Shairella*. *Japonitatahoujayi***sp. nov.** and *J.jungchani***sp. nov.** are described. Biological information is provided for *J.houjayi***sp. nov.** In addition, the generic boundary of *Shairella* is redefined by including *S.quadricostata* and *S.caerulea*.

## ﻿Introduction

Weise (1922) described a new genus, *Japonia* Weise, 1922, based on the species *Phyllobroticanigrita* Jacoby, 1886. However, *Japonia* a junior homonym of a snail genus (Gould 1859). A replacement name, *Japonitata*, was proposed by Strand (1935). This genus is composed more than 30 species in the Oriental and Palaearctic regions (Nie et al. 2017). Most species (90%) were described after 1980 and 60% were described from China (Kimoto 1970, 1996, 2004; Chen and Jiang 1981, 1986; Jiang 1989; Yang 1992; Yang et al. 1997; Medvedev and Sprecher-Uebersax 1998, 1999; Yang and Li 1998; Medvedev 2012). Although most species were described during the past 50 years, none or few morphological characters were illustrated or included in species descriptions.

In Taiwan, only two species were described by Kimoto (1996), with no additional information provided since then. The Taiwan Chrysomelid Research Team (TCRT) was founded in 2005 and is composed of ten members. All of them are amateurs interested in producing a complete inventory of chrysomelid species in Taiwan. Members of TCRT have collected sufficient material of the two Taiwanese species of *Japonitata* to allow their biology to be explored. These two species are different not only in color forms (red vs. black) and behavior (diurnal vs. nocturnal), but also in morphology. Nocturnal species shows great similarity to species of *Shairella* Chûjô, 1962. Two more species are now available for study with help from citizen scientists and loans from museums. In addition, *Japonitata* Strand is similar to *Paraplotes* Laboissière and some species of both genera have been confused. For example, *J.clavata* Yang & Wu, 1998 is a junior synonym of *P.clavicornis* Gressitt & Kimoto, 1963 and *P.rugatipennis* (Chen & Jiang, 1986) was transferred from *Japonitata* by Zhang et al. (2008); *J.indica* (Takizawa & Basu, 1987) was transferred from *Paraplotes* by Medvedev (2002). Diagnostic characters proposed by Zhang et al. (2008) for both genera are evaluated here. In this study, besides describing new species and redescribing known species, the taxonomic status of these is evaluated by redefining the genus *Japonitata* and its allied genera, *Paraplotes* and *Shairella*.

## ﻿Materials and methods

For taxonomic study, the abdomens of adults were separated from the forebodies and boiled in 10% KOH solution, followed by washing in distilled water to prepare genitalia for illustrations. The genitalia were then dissected from the abdomens, mounted on slides in glycerin, and studied and drawn using a Leica M165 stereomicroscope. For detailed examinations a Nikon ECLIPSE 50i microscope was used.

At least three pairs from each species were examined to delimit variability of diagnostic characters. For species collected from more than one locality, at least one pair from each locality was examined. Length was measured from the anterior margin of the eye to the elytral apex, and width at the greatest width of the elytra.

Specimens studied herein are deposited at the following institutes and collections:

**NMNS**National Museum of Natural Science, Taichung, Taiwan [Jing-Fu Tsai]

**OMNH**Osaka Museum of Natural History, Osaka, Japan [Shunpei Fujie]

**SEHU**Laboratory for Systematic Entomology, Hokkaido University, Sapporo, Japan [Masahiro Ohara]

**TARI**Applied Zoology Division, Taiwan Agricultural Research Institute, Taichung Taiwan

Exact label data are cited for all type specimens of described species; a double slash (//) divides the data on different labels and a single slash (/) divides the data in different rows. Other comments and remarks are in square brackets: [p] – preceding data are printed, [h] – preceding data are handwritten, [w] – white label, [y] – yellow label, and [r] – red label.

For redefining the genus *Japonitata*, specimens of the type species, *J.nigrita*, were studied: 1♀ (OMNH), 春日山, Nara Pref., 11.VI.1968, leg. O. Tominaga; 1♀ (OMNH), Hirakura, Mie Univ. Forest, 7.VII.1954, leg. Z. Naruse.

## ﻿Taxonomy

### ﻿ Japonitata

Taxon classificationAnimaliaColeopteraChrysomelidae

Strand, 1935

1D5C9391-D543-54E3-AEC3-35ED77810772

Japonia Weise, 1922: 70 (Type species: Phyllobroticanigrita Jacoby, 1885).
Japonitata Strand, 1935: 294 (replacement name for Japonia Weise, 1922 nec Gould, 1859).

#### Diagnosis.

*Japonitata* can be separated from *Paraplotes* by the presence of posteriorly open anterior coxal cavities (closed in *Paraplotes*); pronotum longer, 1.5–1.7 × wider than long (pronotum short, 2.4–2.9 × wider than long in *Paraplotes*), basal border immarginate (basal border margined in *Paraplotes*); disc with lateral depressions (disc with transverse depressions in *Paraplotes*); disc of elytra with reduced punctures (disc of elytra with fine or coarse punctures in *Paraplotes*), with one more longitudinal ridge in addition to lateral ridge. Other characters proposed by Zhang et al. (2008) are not diagnostic. Antennae are variable among *Paraplotes* species. For example, ratios of length to width from antennomeres I–XI of males of *P.taiwana* Chûjô, 1963: 3.2: 1.6: 2.4: 2.8: 2.8: 2.1: 2.3: 2.2: 2.9: 3.1: 4.6; antennomeres VI–VIII much shorter than those of *J.jungchani* sp. nov., but much narrower in those of *P.cheni* Lee, 2015 (sympatric with *P.taiwana*), ratios of length to width from antennomeres I–XI of males 3.3: 1.6: 3.1: 3.3: 3.5: 3.1: 3.4: 3.7: 3.6: 3.9: 5.0. These characters are not diagnostic for either genus. The rugose or pubescent disc of the elytra occurs in some species of *Paraplotes*. Thus, it is not diagnostic. Appendiculate tarsal claws occur in both genera, with no difference between them. Some genitalic characters are diagnostic. Aedeagi of adults of *Japonitata* have a well sclerotized, elongate tectum (variable tectum with one pair of apico-lateral sclerites in *Paraplotes*), lacking endophallic spicula (with one long median spiculum, and one or two additional pairs of lateral spicula in *Paraplotes*); spermathecal receptaculum as wide as pump (spermathecal receptaculum swollen, wider than pump in *Paraplotes*).

*Japonitata* species are also similar to those of *Shairella* with the lateral borders of pronotum marginate but apical and basal borders unmargined. However, *Japonitata* differs from *Shairella* by the posteriorly open anterior coxal cavities (closed in *Shairella*); robust antennae, antennomeres IV–X less than 3.5 × longer than wide (antenna slender, antennomeres IV–X more than 3.5 × longer than wide in *Shairella*), with distinct lateral ridges and an additional longitudinal, distinct ridge on each elytron (with weak lateral ridge and no additional distinct ridge on each elytron in *Shairella*). Aedeagi of adults of *Japonitata* have a well sclerotized, elongate tectum (membranous tectum in *Shairella*); lack endophallic spicula (with one slender median speculum in *Shairella*); spermathecal receptaculum short, wider than pump (spermathecal receptaculum long, as wide as pump in *Shairella*). Diagnostic characters of *Japonitata*, *Paraplotes*, and *Shairella* can be summarized as follows (Table 1).

**Table 1.** Diagnostic character states for *Japonitata*, *Paraplotes*, and *Shairella*.

Characters / Genera	*Japonitata*	*Paraplotes*	*Shairella*
Antennae	Robust, antennomeres VI–X less than 4.0 × longer than wide	Robust, antennomeres VI–X less than 4.0 × longer than wide	Slender, antennomeres VI–X less than 4.0 × longer than wide
Anterior coxal cavities	Open posteriorly	Closed	Closed
Basal border of pronotum	Unmargined	Margined	Unmargined
Depression on pronotum	Interrupted from middle	Continuous	Interrupted from middle
Shape of pronotum	1.5–1.7 × wider than long	Short, transverse, 2.4–2.9 × wider than long	1.8–2.2 × wider than long
Ridges on elytra	Lateral ridge distinct, with one more longitudinal, distinct ridge	Lateral ridge distinct, no additional longitudinal ridges	Lateral ridge weak, without additional longitudinal ridges
Punctures on elytra	Reduced	Fine or coarse	Reduced or fine
Median internal ridge on abdominal ventrite V in males	Starting from base	Reduced	Starting from apex
Tectum of aedeagus	Well sclerotized, elongate	Variable, and with one pair of apico-lateral sclerites	Membranous
Endophallic sclerites	None	One median, longitudinal spiculum without clustered short setae, and one or two pairs of lateral sclerites	Only one median, longitudinal spiculum with clustered short setae
Spermathecal receptaculum	Swollen and short, wider than pump	Narrow and short, as wide as pump	Narrow and long, as wide as pump
Behavior	Diurnal	Nocturnal or Diurnal	Nocturnal

#### Remarks.

*Japonitataquadricostata* Kimoto, 1996 and *J.caerulea* Kimoto, 1996 are transferred to *Shairella* since both species fit the redefinition of the genus. They are characterized by normal elytra. Shortened elytra and reduced hindwings occur in all other species of *Shairella*; however, reduced hindwings also occur in some populations of *S.quadricostata*.

#### Included species.

More than 30 species are distributed in Oriental and Palaearctic regions (Nie et al. 2017) but their taxonomic status should be re-evaluated since two species are transferred to *Shairella*, and others may also require transfer.

### ﻿ Japonitata houjayi sp. nov.

Taxon classificationAnimaliaColeopteraChrysomelidae

808114EC-E8D1-5A35-8E87-15D370620AC8

https://zoobank.org/68D59E89-7532-41EA-AB82-B493814CD51B

Figs 1A–C , 2 , 3

#### Types.

***Holotype*** ♂ (TARI). Taipei, Shihtzutoukeng (獅子頭坑), 300 m, 1.V.2010, leg. H.-J. Chen. ***Paratypes***: 1♂, 5♀ (TARI), same data as holotype; 4♂, 1♀ (TARI), same but with “4.V.2010”; 4♂, 3♀ (TARI), same but with “8.V.2010”; 1♂, 1♀ (TARI), same but with “26.V.2010”; 3♂, 4♀ (TARI), same but with “28.V.2010”; 3♂ (TARI), same but with “leg. H. Lee”; 1♂ (TARI), same locality, 25.IV.2012, leg. H.-J. Chen.

#### Description.

**Figure 1.**
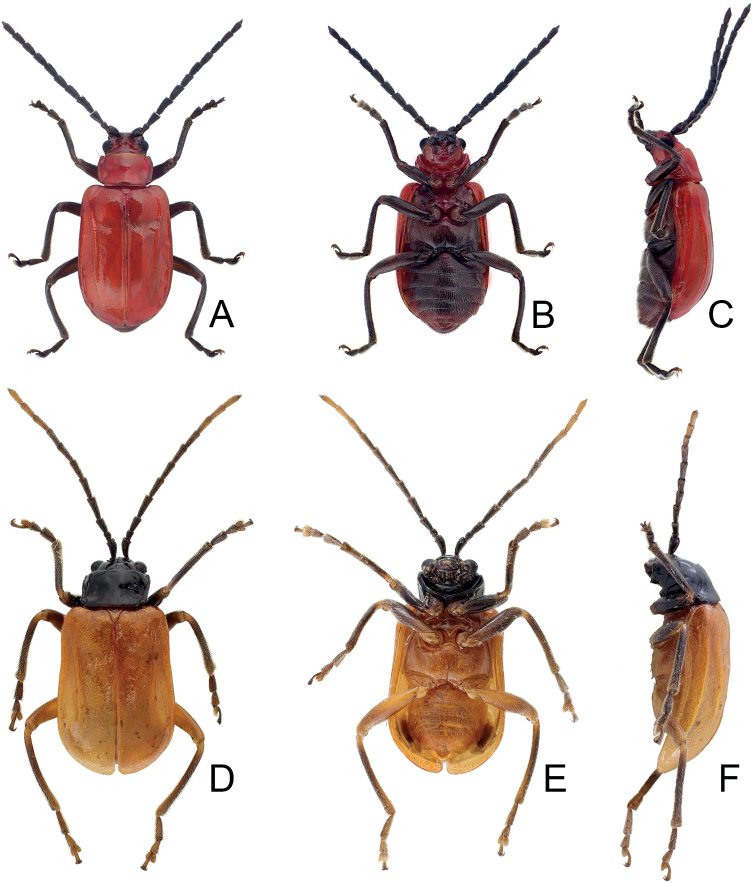
Habitus of *Japonitatahoujayi* sp. nov. and *J.jungchani* sp. nov. **A***J.houjayi* sp. nov., male, dorsal view **B** ditto, ventral view **C** ditto, lateral view **D***J.jungchani* sp. nov., male, dorsal view **E** ditto, ventral view **F** ditto, lateral view.

**Figure 2.**
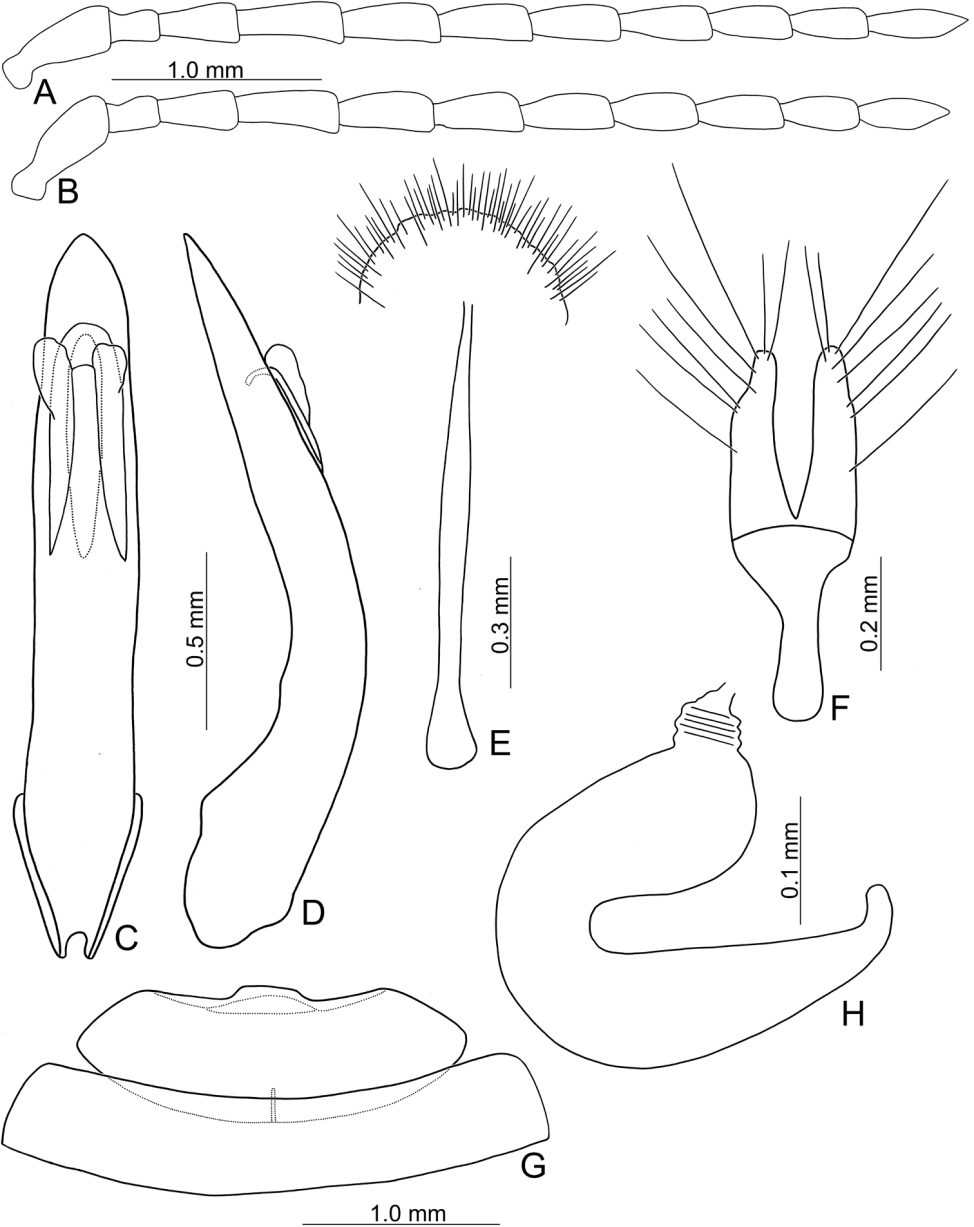
Diagnostic characters of *Japonitatahoujayi* sp. nov. **A** antenna, male **B** antenna, female **C** aedeagus, dorsal view **D** ditto, lateral view **E** abdominal ventrite VIII **F** gonocoxae **G** abdominal ventrite IV–V, male **H** spermatheca.

Length 5.5–6.6 mm, width 2.7–3.4 mm. General color (Fig. 1A–C) reddish brown; antennae black; legs dark brown. Antennomeres II–XI filiform but stout in males (Fig. 2A), ratios of lengths of antennomeres I–XI 1.0: 0.4: 0.7: 0.9: 0.8: 0.8: 0.8: 0.8: 0.7: 0.7: 0.8; ratios of length to width from antennomeres I–XI 2.5: 1.5: 1.9: 2.7: 2.5: 2.5: 2.5: 2.5: 2.4: 2.4: 3.5; stout antennae in males similar in females (Fig. 2B), ratios of lengths of antennomeres I–XI 1.0: 0.4: 0.6: 0.8: 0.8: 0.7: 0.8: 0.7: 0.7: 0.6: 0.8; ratios of length to width from antennomeres I–XI 2.5: 1.5: 2.0: 2.5: 2.4: 2.3: 2.5: 2.4: 2.6: 2.3: 2.9. Pronotum 1.6–1.7 × wider than long; disc with scarce, fine punctures at sides, reduced medially, with transverse groove near base, medially abbreviated, laterally connected with short longitudinal groove on basal margin; lateral margins slightly rounded, widest behind apices; apical margin slightly concave and basal margin slightly convex. Elytra 1.5 × longer than wide; disc with confused, dense, reduced punctures; with one small tubercle behind scutellum; with one distinct longitudinal ridge from humeral calli, parallel with lateral margin, abbreviated subapically; with one additional ridge also from humeral calli, distinct, directed medially; lateral margins moderately rounded, widest at apical third, apices convergent. Aedeagus (Fig. 2C, D) extremely slender, 7.5 × longer than wide; parallel-sided, slightly narrowed at apical 1/4, strongly narrowed subapically, apex narrowly rounded; moderately curved at basal 1/3 in lateral view; tectum slender, longitudinal, apex recurved; no endophallic sclerites. Apical margin of abdominal ventrite V in males with distinct, narrow median lobe (Fig. 2G), apical margin slightly recurved, with short median internal ridge at middle of basal margin, from basal fourth to base; basal margin normal. Gonocoxae (Fig. 2F) longitudinal and connected basally, with narrow furrow between gonocoxae; each gonocoxa narrowed subapically, apex narrowly rounded, with eight long apical setae; base strongly sclerotized and narrow. Ventrite VIII (Fig. 2E) in females with apex weakly sclerotized, small, apical margin irregular; with dense short apical setae; spiculum extremely elongate. Spermathecal receptaculum (Fig. 2H) swollen, not delimited from pump; pump long and curved, with apical process curved; sclerotized spermathecal duct extremely short, not separated from receptaculum.

#### Diagnosis.

Adults of *J.houjayi* sp. nov. are similar to those of *J.ruficollis* Jiang, 1989 from China (Xizang) with reddish brown bodies, but differ in possessing black antennae and dark brown legs (yellow antenna with one or two apical antennomeres black, and reddish brown legs in *J.ruficollis*).

#### Host plant.

*Scutellariaindica* L. (Lamiaceae).

#### Biology.

**Figure 3.**
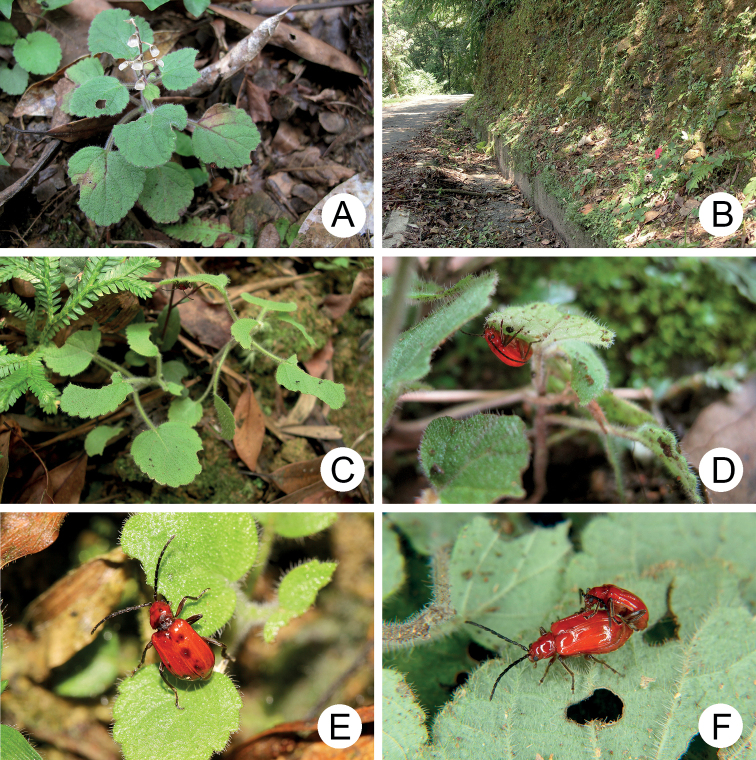
Field photographs of *Japonitatahoujayi* sp. nov. **A** host plant, *Scutellariaindica***B** population of *Scutellariaindica* growing on the slope along a road **C** adult resting on underside of leaf **D** close-up of adult **E** adult feeding on leaf **F** adults mating on a leaf.

*Scutellariaindica* is a small herbaceous plant (Fig. 3A) growing on slopes along roads (Fig. 3B). Adults appear only during May, usually resting on the undersides of leaves during daytime (Fig. 3C, D). Adults feed on the leaves (Fig. 3E) and were observed mating (Fig. 3F) occasionally.

#### Etymology.

The new species name is dedicated to Mr. Hou-Jay Chen (陳厚潔), the first team member to find the habitat and collect type specimens.

#### Distribution.

This new species is known only from the type locality.

### ﻿ Japonitata jungchani sp. nov.

Taxon classificationAnimaliaColeopteraChrysomelidae

98411A08-CF77-55CF-991A-DC4D795AF174

https://zoobank.org/9E6F19B8-0E5B-4E46-B346-2E2F8962A2C4

Figs 1D–F , 4

#### Types.

***Holotype*** ♂ (TARI), Pingtung, Tahanshan (大漢山), 1450 m, 12.IV.2020, leg. Y.-T. Chung. ***Paratypes*.** 1♀ (TARI), same locality, 4.IV.2010, leg. K.-D. Ho; 1♀ (TARI), same locality (= Jinshuiying 浸水營), 6.VI.2011, leg. J.-C. Chen; 1♂ (TARI), same but with “22.V.2012”; 1♀ (TARI), Taitung, Lichia trail (利嘉林道), 1000 m, 10.V.2018, leg. B.-X. Guo.

#### Description.

**Figure 4.**
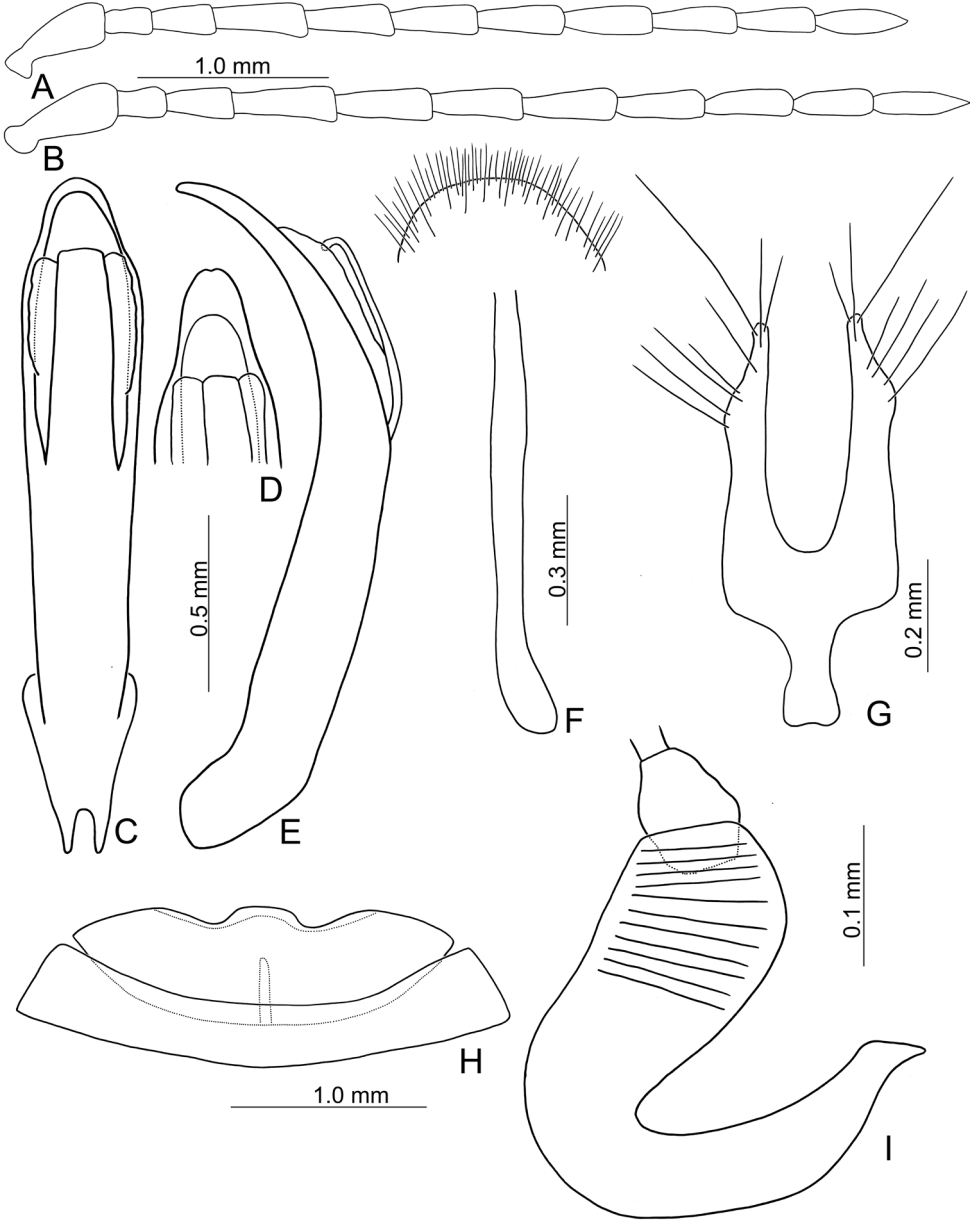
Diagnostic characters of *Japonitatajungchani* sp. nov. **A** antenna, male **B** antenna, female **C** aedeagus, dorsal view **D** apex of aedeagus, front view **E** aedeagus, lateral view **F** abdominal ventrite VIII **G** gonocoxae **H** abdominal ventrite IV–V, male **I** spermatheca.

Length 5.8–6.3 mm, width 3.1–3.3 mm. General color (Fig. 1D–F) reddish brown; head and prothorax black; legs dark brown. Antennomeres II–XI filiform but stout in males (Fig. 4A), ratios of lengths of antennomeres I–XI 1.0: 0.4: 0.6: 0.8: 0.8: 0.8: 0.8: 0.8: 0.8: 0.7: 0.8; ratios of length to width from antennomeres I–XI 2.5: 1.7: 2.1: 2.8: 2.6: 2.9: 2.9: 3.2: 3.2: 3.1: 3.9; stout antennae in males similar in females (Fig. 4B), ratios of lengths of antennomeres I–XI 1.0: 0.4: 0.5: 0.8: 0.8: 0.8: 0.8: 0.7: 0.7: 0.7: 0.8; ratios of length to width from antennomeres I–XI 2.7: 1.6: 2.0: 3.0: 3.1: 3.0: 3.1: 3.1: 3.2: 2.9: 4.0. Pronotum 1.5–1.6 × wider than long; disc with scarce, fine punctures at sides, reduced medially, with transverse groove near base, medially abbreviated, laterally connected with short longitudinal groove on basal margin; lateral margins slightly rounded, widest behind apices; apical margin slightly concave and basal margin slightly convex. Elytra 1.4 × longer than wide; disc with confused, dense, fine punctures; with one small tubercle behind scutellum; with one distinct longitudinal ridge from humeral calli, parallel with lateral margin, abbreviated subapically; with one additional ridge also from humeral calli, distinct, directed medially; lateral margins moderately rounded, widest at apical third, apices divergent. Aedeagus (Fig. 4C–E) extremely slender, 6.4 × longer than wide; widest at apical 1/6, gradually narrowed toward base, moderately narrowed at apical 1/6, apex widely rounded, slightly and medially depressed; strongly, apically curved in lateral view; tectum wide, longitudinal, apex recurved; no endophallic sclerites. Apical margin of abdominal ventrite V in males with distinct median lobe (Fig. 4H) narrow, apical margin slightly recurved, with long median internal ridge at middle of basal margin, from base to middle; basal margin normal. Gonocoxae (Fig. 4G) longitudinal and connected basally, with narrow furrow between gonocoxae; each gonocoxa narrowed subapically, apex narrowly rounded, with eight long apical setae; base weakly sclerotized and narrow. Ventrite VIII (Fig. 4F) in females with apex weakly sclerotized, small, apical margin slightly irregular; with dense short apical setae; spiculum extremely elongate. Spermathecal receptaculum (Fig. 4I) swollen, not separated from pump; pump long and curved, with apical process curved; sclerotized spermathecal duct extremely short, separated from receptaculum.

#### Diagnosis.

This new species is similar to *J.bipartita* Chen & Jiang, 1986 from China (Shaanxi and Fujian) with reddish brown body and black head and prothorax. It differs in having black antenna with the three apical antennomeres reddish brown, and dark brown fore and middle legs.

#### Host plant and biology.

Unknown, but one adult was collected by sweeping flowers.

#### Etymology.

The new species name is dedicated to Mr. Jung-Chan Chen (陳榮章), the first person to collect type specimens.

#### Distribution.

South Taiwan including Pingtung and Taitung counties.

### ﻿ Shairella quadricostata

Taxon classificationAnimaliaColeopteraChrysomelidae

(Kimoto, 1996) comb. nov.

82C415DF-B2AD-5548-B1D3-0A8B6AAE60E4

Figs 5 , 6 , 7 , 8

Japonitata quadricostata Kimoto, 1996: 34 (Taiwan).

#### Type examined.

**Figure 5.**
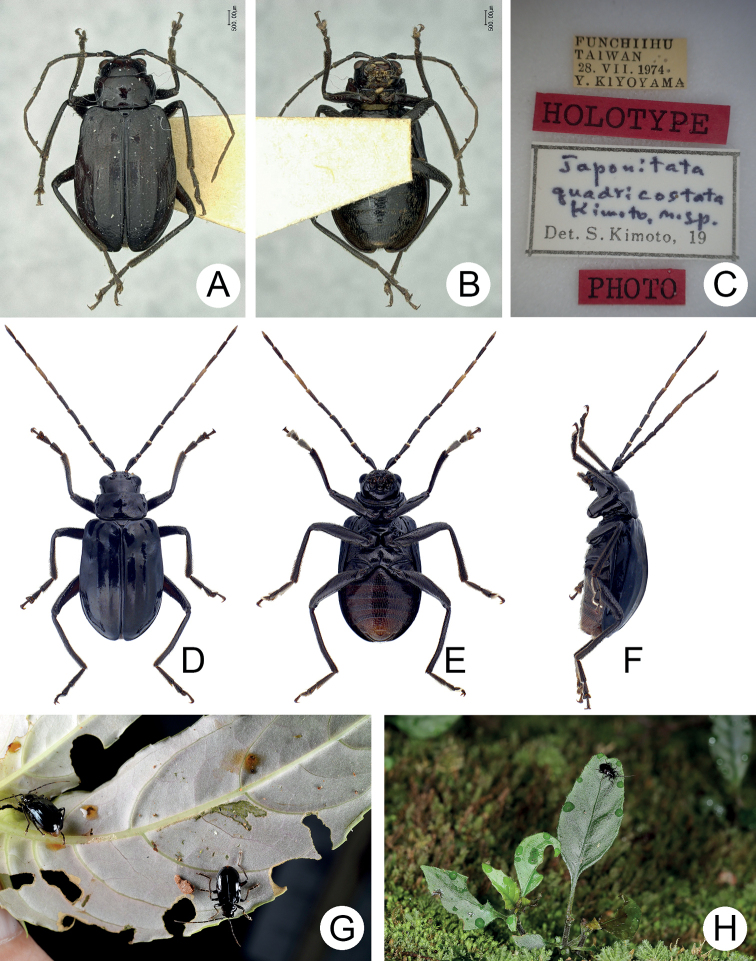
Habitus and field photographs of *Shairellaquadricostata* (Kimoto) **A** holotype, female, dorsal view **B** ditto, lateral view **C** labels on the holotypes **D** nontype, male, dorsal view **E** ditto, ventral view **F** ditto, lateral view **G** two adults collected at Tengchih (藤枝) and feeding on leaves of *Hemiboeabicornuta***H** adult resting on leaves of *Hemiboeabicornuta* in Erhwanping (二萬坪).

***Holotype*** ♀ (OMNH) (Fig. 5A–C): “FUNCHIIHU (奮起湖) / TAIWAN / 28.VII.1974 / Y. KIYOYAMA [p, y] // HOLOTYPE [p, r] / Japonitata / quadricostata / Kimoto, n. sp. [h] / Det. S. Kimoto, 19 [p, w] // PHOTO [p, r]”.

#### Specimens examined.

**Chiayi**: 28♂, 11♀ (TARI), Erhwanping (二萬坪), 2000 m, near Alishan (阿里山), 9.VII.2014, leg. C.-F. Lee & T.-H. Lee; 1♂ (TARI), Alishan (阿里山), 17.VIII.2014, leg. B.-X. Guo; **Ilan**: 1♂ (TARI), Chiuchihtse (鳩之澤), 520 m, 2.V.2007, leg. M.-H. Tsou; 1♂ (TARI), Eboshiyama (= Tulishan 獨立山), 1900 m, 17–21.V.1933, leg. M. Chujo; **Kaohsiung**: 1♂, 1♀ (TARI), Tengchih (藤枝), 1600 m, 24.VIII.2017, leg. B.-X. Guo; 1♂ (TARI), same but with “4.IX.2017”; 1♀ (TARI), same but with “15.IX.2019”; 3♂ (TARI), same locality, 11.V.2022, leg. Y.-T. Chung; **Nantou**: 2♀ (TARI), Fenghuangshan (鳳凰山), 1700 m, near Hsitou (溪頭), 12.VIII.2010, leg. Y.-T. Wang; 1♂ (TARI), Hsitou (溪頭), 1000 m, 14.VI.2011, leg. C.-F. Lee; 4♀ (TARI), same locality, 2.VII.2011, leg. M.-H. Tsou; 1♂, 1♀ (TARI), same but with “9.VIII.2011”; **Pingtung**: 1♂ (TARI), Peitawushan (北大武山), New Trailhead (新登山口), 1200 m, 28.IX.2017, leg. Y.-T. Chung; 1♂ (TARI), same but with “10.V.2022”; 1♂ (TARI), Shuangliu (雙流), 500 m, 6.V.2000, leg. H.-T. Shih; **Taichung**: 1♀ (TARI), Fengyuan (豐原), 280 m, 22.V.2019, leg. C.-T. Hsu; 1♂ (TARI), Henglingshan (橫嶺山), Trailhead (登山口), 1200 m, 10.X.2020, leg. Y.-C. Hsu; **Taipei**: 1♂ (TARI), Manyuehyuan (滿月圓), 300 m, 7.VI.2010, leg. C.-L. Chiang; 1♀ (TARI), Wulai (烏來), 150 m, 24.V.2007, leg. H.-J. Chen; 1♂, 1♀ (TARI), same locality (= Hsinhsien 信賢), 3.V.2014, leg. M.-H. Tsou.

#### Redescription.

**Figure 6.**
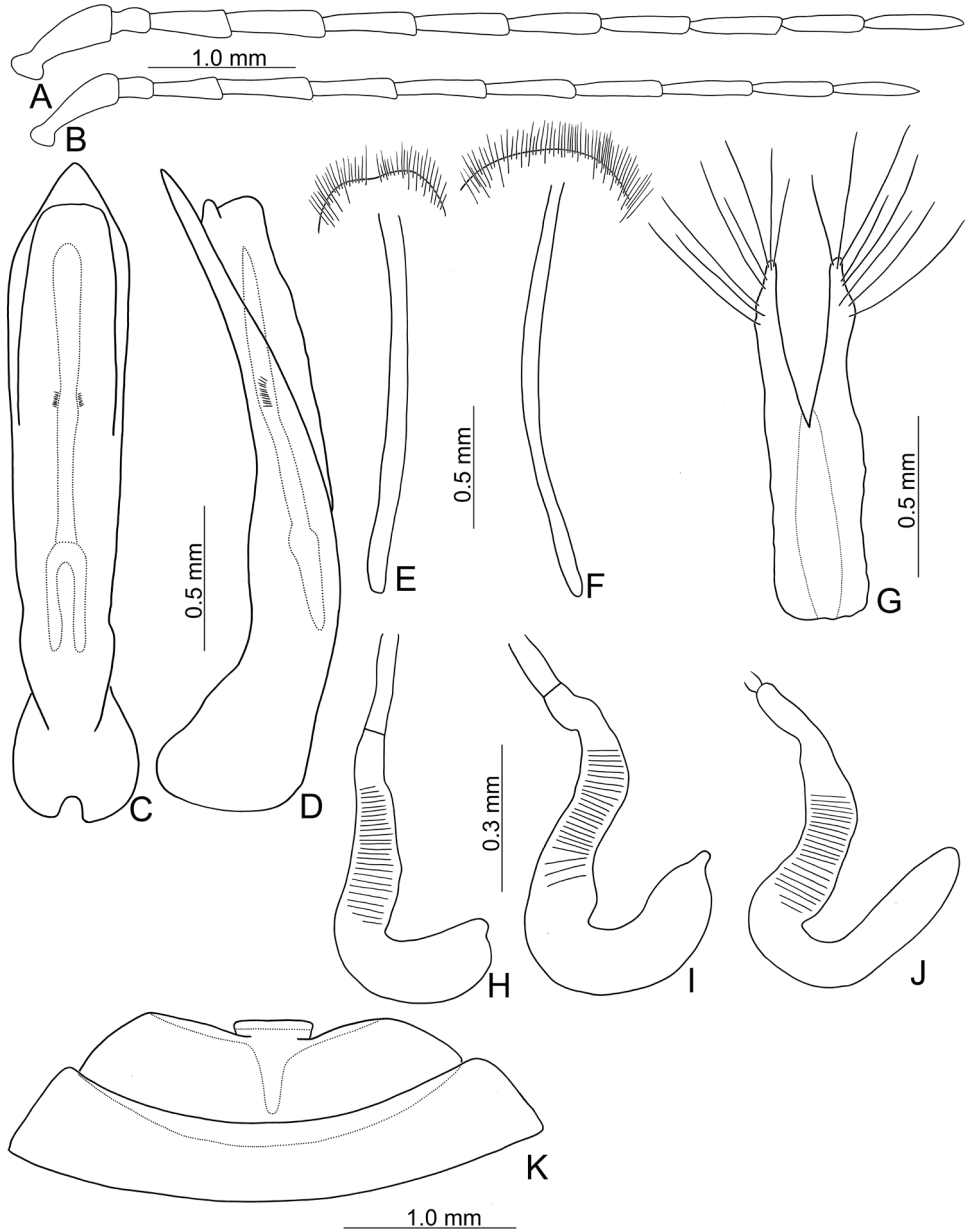
Diagnostic characters of *Shairellaquadricostata* (Kimoto) **A** antenna, male **B** antenna, female **C** aedeagus, dorsal view **D** ditto, lateral view **E** abdominal ventrite VIII, from Erhwanping (二萬坪) **F** same, from Wulai (烏來) **G** gonocoxae **H** spermatheca, from Tengchih (藤枝) **I** same from Wulai (烏來) **J** same from Erhwanping (二萬坪) **K** abdominal ventrite IV–V, male.

Length 6.1–7.7 mm, width 3.1–4.4 mm. General color (Fig. 5D–F) black to dark brown; abdomen yellow to dark brown; five apical antennomeres variably paler. Antennomeres II–XI filiform in males (Fig. 6A), ratios of lengths of antennomeres I–XI 1.0: 0.3: 0.7: 0.9: 0.8: 0.8: 0.8: 0.8: 0.8: 0.7: 0.9; ratios of length to width from antennomeres I–XI 2.8: 1.6: 2.8: 3.8: 4.0: 4.2: 4.5: 4.9: 4.9: 4.8: 6.3; more slender in females (Fig. 6B), ratios of lengths of antennomeres I–XI 1.0: 0.3: 0.6: 0.9: 0.8: 0.8: 0.8: 0.8: 0.8: 0.8: 0.8; ratios of length to width from antennomeres I–XI 3.4: 1.6: 2.9: 4.1: 4.1: 4.9: 5.2: 5.5: 6.1: 6.0: 6.5. Pronotum 1.8–2.0 times wider than long; disc with scarce fine punctures at sides, reduced medially, with transverse groove near base, medially abbreviated, laterally connected with short longitudinal groove on basal margin; lateral margins slightly rounded, widest behind apices; apical margin slightly concave and basal margin slightly convex. Elytra narrower, 1.3–1.4 times longer than wide; disc with confused, sparse, reduced punctures; with one small tubercle behind scutellum; with one longitudinal ridge behind tubercle, indistinct, close to suture; with one additional longitudinal ridge outside tubercle, indistinct; with one additional distinct ridge from humeral calli, parallel with lateral margin, abbreviated subapically; another additional ridge also from humeral calli, indistinct, directed medially; lateral margins moderately rounded, widest at apical third, apices convergent. Aedeagus (Fig. 6C, D) slender, 5.9 × longer than wide; lateral margins straight, widest at apical 1/10, gradually narrowed toward basal 1/3; strongly narrowed subapically, apex acute; moderately curved in lateral view; tectum membranous; one endophallic sclerite longitudinally oriented and slender, 0.6 × as long as aedeagus, base deeply bifurcate, lateral margins with clustered short setae at apical 1/3. Apical margin of abdominal ventrite V in males with distinct median lobe (Fig. 6K), narrow, apical margin slightly recurved, with median internal ridge from apex to middle; basal margin normal. Gonocoxae (Fig. 6G) longitudinal and connected basally, with wide furrow between gonocoxae; each gonocoxa narrowed subapically, apex truncate, with eight long apical setae; base weakly sclerotized. Ventrite VIII (Fig. 6E) in females with apex weakly sclerotized, dense short apical setae, reduced medially; spiculum extremely elongate. Spermathecal receptaculum (Fig. 6H) slender, as wide as pump, not separated from pump; pump long and curved, with one short, apical process; sclerotized spermathecal duct short, not separated from receptaculum.

#### Variations.

**Figure 7.**
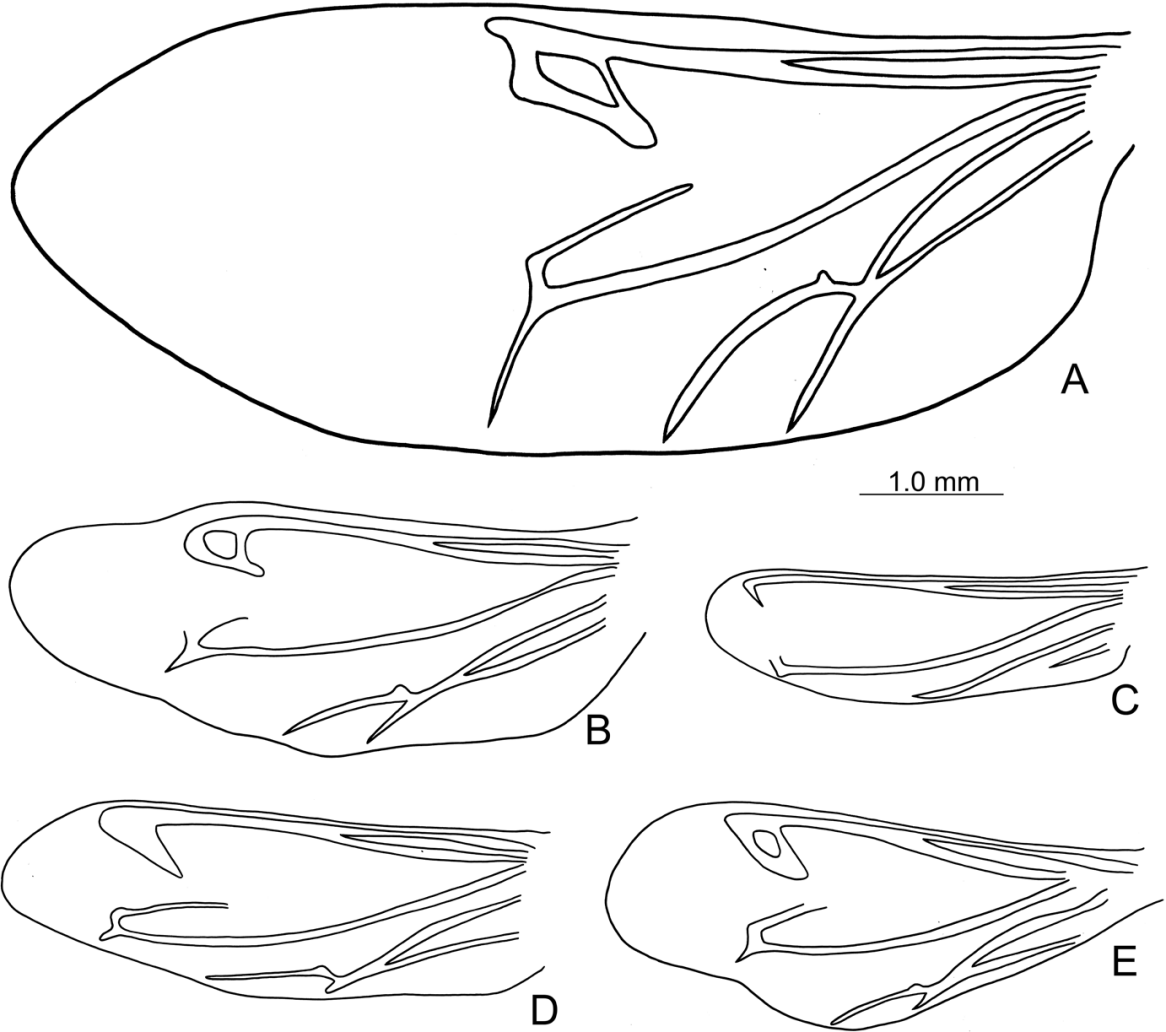
Hindwings of *Shairellaquadricostata* (Kimoto) **A** female, from Wulai (烏來) **B** female, from Tengchih (藤枝) **C** female, from Erhwanping (二萬坪) **D** female, from Hsito (溪頭) **E** male, from Peitawushan (北大武山).

Some distinct variation occurs in female genitalic characters among different populations. Pumps of spermathecae are larger in those of Wulai (烏來) (Fig. 6I); much slender and lacking apical process in those of Erhwanping (二萬坪) (Fig. 6J). Apices of ventrite VIII are wider and setae not reduced medially in those of Wulai (烏來). Hindwings are normal in northern and central Taiwan and low-elevations of southern Taiwan (Fig. 7A), but they are reduced in different degrees between different populations of mid-elevations of southern Taiwan. Degree of reduction of hind wings is similar between individuals of both sexes of the same populations. Those in Tengchih (藤枝) are less reduced, ~ 57% with normal hind wings (Fig. 7B). Those in Hsito (溪頭) are reduced moderately, ~ 50% with normal hind wings (Fig. 7D). Those in Peitawushan (北大武山) have the same length of hind wings as those in Hsito but wider (Fig. 7E). Those in Erhwanping (二萬坪) are reduced strongly, ~ 40% with normal hind wings (Fig. 7C).

#### Diagnosis.

Adults of *Shairellaquadricostata* (Kimoto, 1996), comb. nov. and *S.caerulea* (Kimoto, 1996), comb. nov. are characterized by normal elytra and functional hindwings (shortened elytra and reduced hindwings in other *Shairella*; Lee and Beenen 2017) although individuals in some populations of *S.quadricostata* have more or less reduced hindwings. *Shairellaquadricostata* is distinguished from *S.caerulea* by possessing black elytra with three pairs of weak longitudinal ridges (Fig. 5A–F) (bluish black elytra without longitudinal ridges besides lateral ridge in *S.caerulea*; Fig. 9); median internal ridge of abdominal ventrite V in males expanded from apex, abbreviated before base (Fig. 6K) (median internal ridge of abdominal ventrite in males expanded from apex to base in *S.caerulea*; Fig. 10G); apically narrowed apex of aedeagus (Fig. 6C) (bifurcate apex of aedeagus in *S.caerulea*; Fig. 10C); apex of spermatheca rounded with or without small process (Fig. 6H–J) (apex of spermatheca swollen, bifurcate in frontal view in *S.caerulea*; Fig. 10H, I).

#### Host plant.

*Hemiboeabicornuta* (Hayata) Ohwi (Gesneriaceae).

#### Biology.

Adults of *Shairellaquadricostata* were observed active at night and feeding on leaves of *Hemiboeabicornuta*. However, adults were hard to find with the exception of a single event. Three adults were collected on 11 May 2022 in Tengchih (藤枝) (Fig. 5G). We collected 39 adults on 9 July 2014 in Erhwanping (二萬坪). Many host plants were growing on a steep slope and numerous adults were feeding on leaves (Fig. 5H).

#### Distribution.

**Figure 8.**
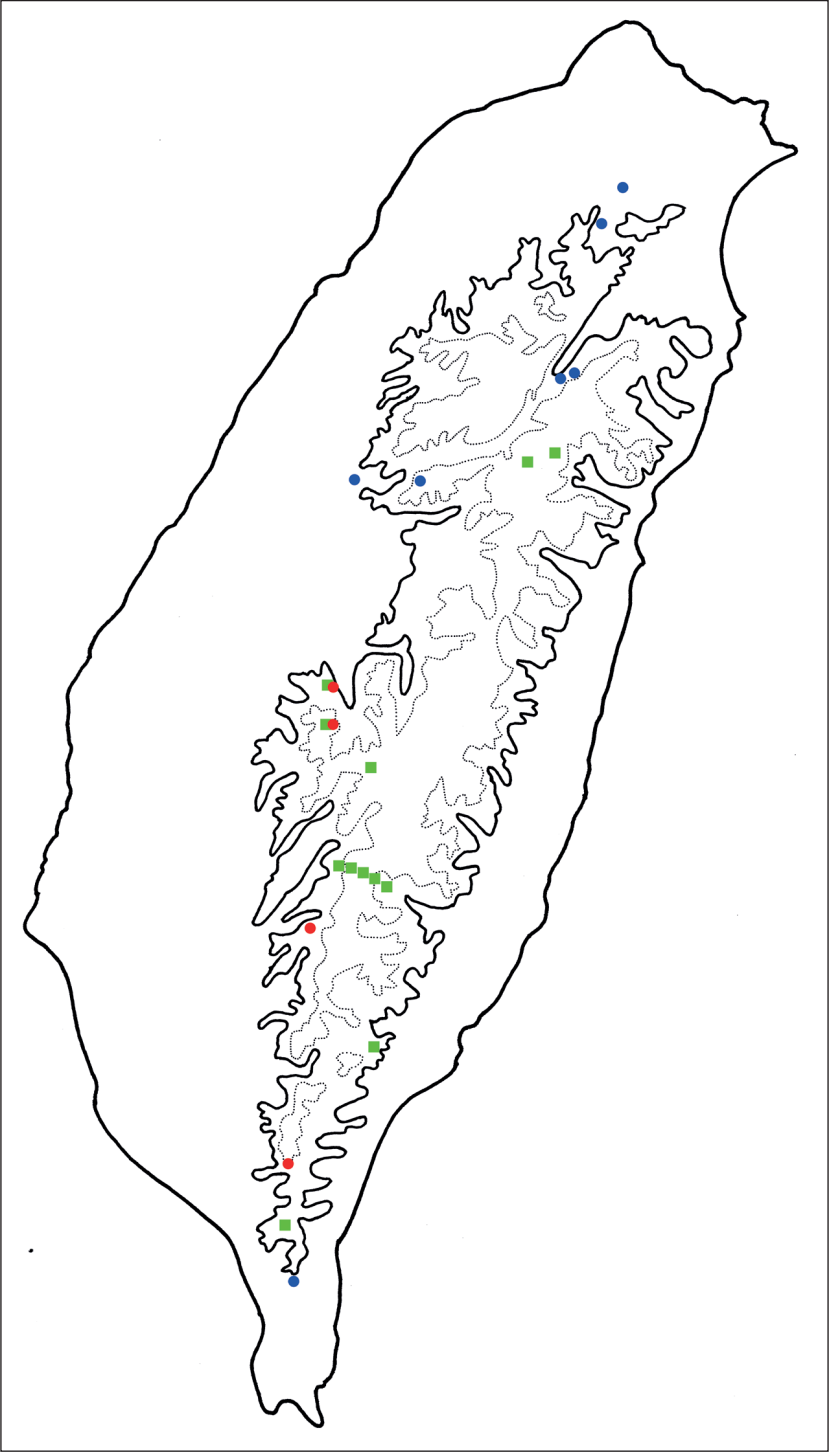
Distribution map of *Shairellaquadricostata* (Kimoto) and brachelytrous *Shairella* species, solid line: 1000 m, broken line: 2000 m. Key: green squares – brachelytrous species, blue circles – adults of *S.quadricostata* with normal hindwings, red circles– adults of *S.quadricostata* with reduced hindwings.

The flighted populations are widespread in low-elevations of Taiwan and mid-elevations of northern and central Taiwan, and flightless populations are restricted to mid-elevations of southern Taiwan (Fig. 8).

### ﻿ Shairella caerulea

Taxon classificationAnimaliaColeopteraChrysomelidae

(Kimoto, 1996) comb. nov.

D34CA586-58B8-5404-B161-27C89FDB02A0

Figs 9 , 10

Japonitata caerulea Kimoto, 1996: 33 (Taiwan).

#### Type examined.

**Figure 9.**
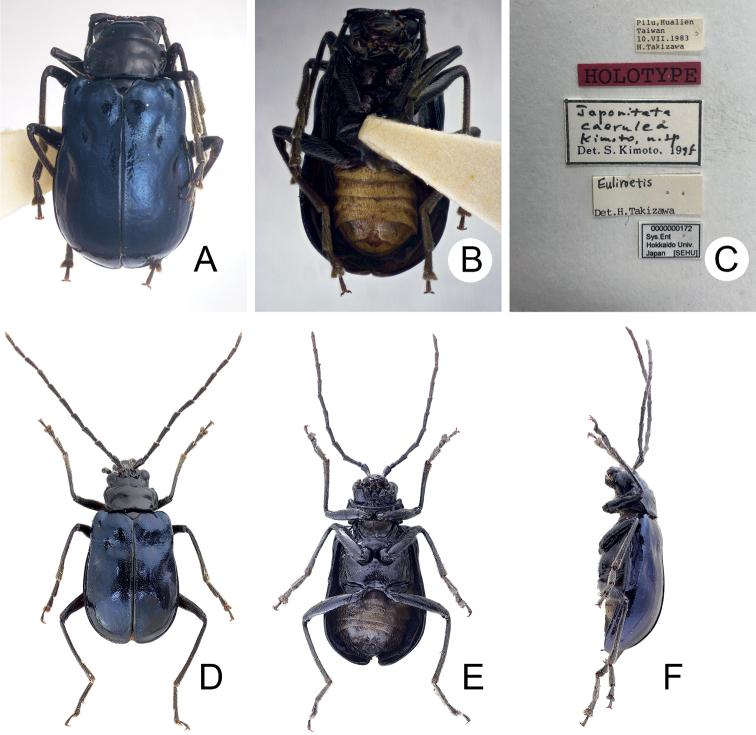
Habitus of *Shairellacaerulea* (Kimoto) **A** holotype, male, dorsal view **B** ditto, lateral view **C** labels on the holotypes **D** nontype, male, dorsal view **E** ditto, ventral view **F** ditto, lateral view.

***Holotype*** ♂ (SEHU) (Fig. 9A–C): “Pilu (碧綠), Hualien / Taiwan / 10.VII.1983 / H. Takizawa [p, w] // HOLOTYPE [p, r] // Japonitata / caerulea / Kimoto, n. sp. [h] / Det. S. Kimoto, 19[p]95[h, w] // Euliroetis [h] / Det. H. Takizawa [p, w] // 0000000172 / Sys. Ent / Hokkaido Univ. / Japan [SEHU] [p, w]”.

#### Specimens examined.

**Hualien**: 1♀ (NMNS), Hualuhsi (華祿溪), 1300 m, 28.VII.–25.IX.2011, leg. W.-T. Yang & K.-W. Huang; 1♀ (NMNS), Biyu Sacred Tree (碧綠神木), 2150 m, 1.VI.–28.VII.2011, leg. W.-T. Yang & K.-W. Huang; 1♂ (NMNS), same but with “28.VII.–5.IX.2011”; 1♂, 1♀ (NMNS), same but with “28.V.–24.VII.2012”; 2♂ (NMNS), same but with “24.VII.–10.IX.2012”; **Kaohsiung**: 1♀ (TARI), Chungchihkuan (中之關), 1930 m, 10.VI.2015, leg. T.-H. Lee; **Nantou**: 1♂ (TARI), Tunyuan (屯原), 1900 m, 21.VI.2019, leg. B.-X. Guo. All specimens from Hualien were collected using Malaise traps.

#### Redescription.

**Figure 10.**
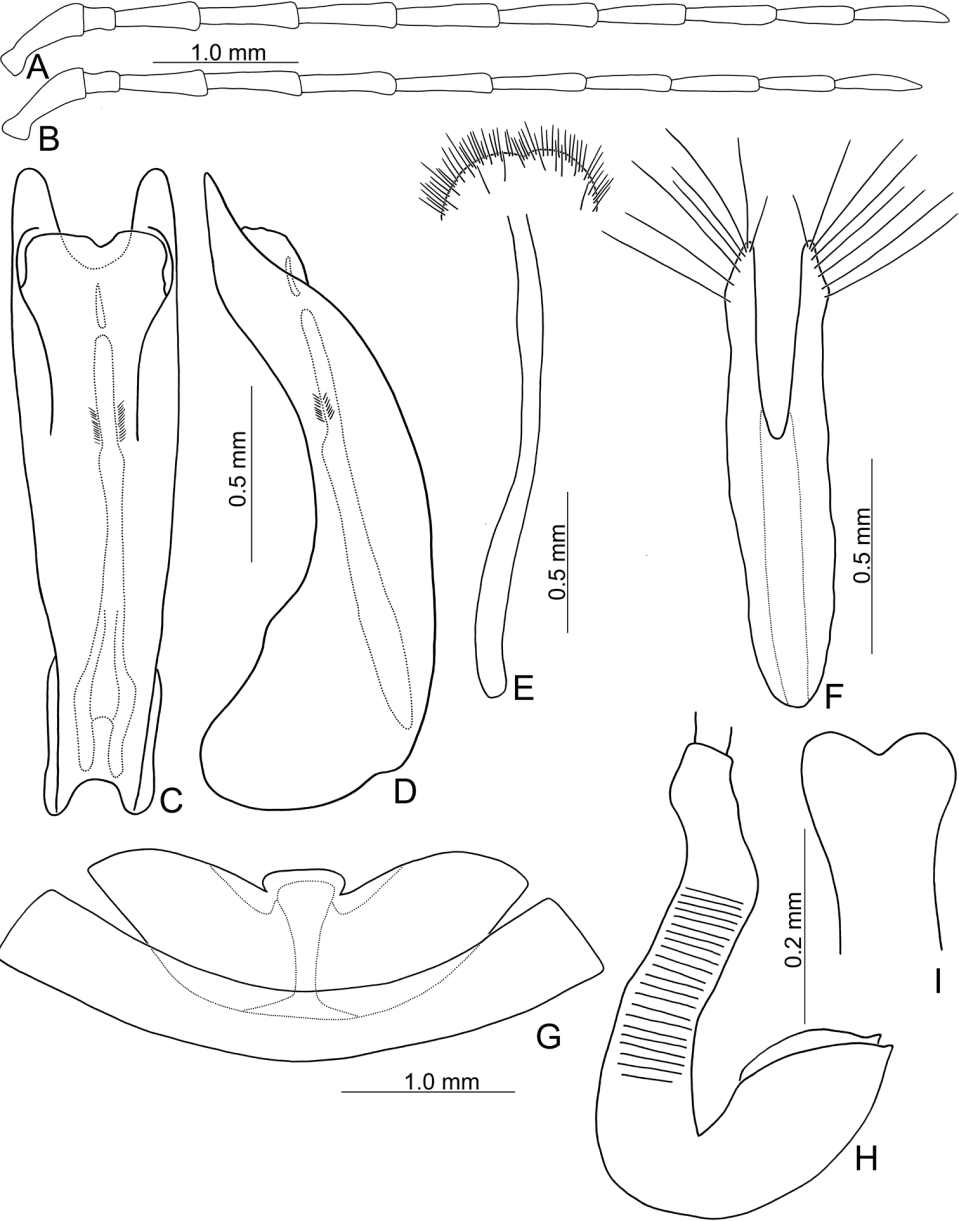
Diagnostic characters of *Shairellacaerulea* (Kimoto) **A** antenna, male **B** antenna, female **C** aedeagus, dorsal view **D** ditto, lateral view **E** abdominal ventrite VIII **F** gonocoxae **G** abdominal ventrite IV–V, male **H** spermatheca **I** apex of spermatheca, front view.

Length 6.8–6.9 mm, width 3.7–3.9 mm. General color (Fig. 9D–F) black to blackish brown; abdomen yellow; elytra bluish black. Antennomeres II–XI filiform in males (Fig. 10A), ratios of lengths of antennomeres I–XI 1.0: 0.3: 0.9: 1.0: 1.1: 1.1: 1.1: 0.9: 0.9: 0.8: 1.0; ratios of length to width from antennomeres I–XI 3.0: 1.4: 2.9: 3.6: 3.9: 4.2: 4.3: 4.2: 4.6: 4.3: 6.1; more slender in females (Fig. 10B), ratios of lengths of antennomeres I–XI 1.0: 0.4: 0.9: 1.0: 1.0: 1.0: 1.0: 0.9: 0.9: 0.8: 0.9; ratios of length to width from antennomeres I–XI 3.0: 1.6: 3.4: 3.9: 4.3: 4.6: 4.8: 5.5: 6.1: 5.3: 6.1. Pronotum 2.2 times wider than long; disc with scarce fine punctures at sides, reduced medially, with transverse groove near base, medially abbreviated, laterally connected with short longitudinal groove on basal margin; lateral margins slightly rounded, widest behind apices; apical margin slightly concave and basal margin slightly convex. Elytra 1.4 × longer than wide; disc with confused, dense, fine punctures; with one small tubercle behind scutellum, with one deep depression behind tubercle; with one indistinct longitudinal ridge from humeral calli, parallel with lateral margin, abbreviated subapically; with one additional, deep depression at middle, above longitudinal ridge; lateral margins moderately rounded, widest at apical third, apices divergent. Aedeagus (Fig. 10C, D) wide, 4.4 × longer than wide; lateral margins straight, widest at apex, gradually narrowed towards base; apex with deep notch; moderately curved in lateral view; tectum membranous; one endophallic sclerite longitudinal and slender, 0.7 × as long as aedeagus, base shallowly bifurcate, lateral margins with clustered short setae at apical third; with short membranous area near apex. Apical margin of abdominal ventrite V in males with distinct median lobe (Fig. 10G), narrow, apical margin slightly recurved, with median internal ridge from apex to base, with narrow furrow between gonocoxae; basal margin expanding posteriorly. Gonocoxae (Fig. 10F) longitudinal and connected basally; each gonocoxa narrowed subapically, apex truncate, with eight long apical setae; base weakly sclerotized but strongly sclerotized medially. Ventrite VIII (Fig. 10E) in females with apex weakly sclerotized, small, depressed medially; with dense short apical setae; spiculum extremely elongate. Spermathecal receptaculum (Fig. 10H, I) slender, as wide as pump, not separated from pump; pump long and curved, apex slightly swollen, dorso-ventrally bifurcate; sclerotized spermathecal duct short, not separated from receptaculum.

#### Diagnosis.

*Shairellacaerulea* (Kimoto, 1996), comb. nov. and *S.quadricostata* (Kimoto, 1996), comb. nov. are characterized by having normal elytra and functional hindwings (shortened elytra and reduced hindwings in other species; Lee and Beenen 2017) although some populations of *S.quadricostata* have variably reduced hindwings. *Shairellacaerulea* is distinguished easily from *S.quadricostata* by its bluish black elytra without longitudinal ridges other than the lateral ridge (Fig. 9) (black elytra with three pairs of weak longitudinal ridges in *S.quadricostata*; Fig. 5); median internal ridge of abdominal ventrite in males expending from apex into base (Fig. 10G) (median internal ridge of abdominal ventrite V in males expanding from apex, abbreviated before base in *S.quadricostata*; Fig. 6K); bifurcate apex of aedeagus (Fig. 10C) (apically narrowed apex of aedeagus in *S.quadricostata*; Fig. 6C); apex of spermatheca swollen, bifurcate in frontal view (Fig. 10H, I) (apex of spermatheca rounded with small process in *S.quadricostata*; Fig. 6H–J).

#### Host plant and biology.

Unknown.

#### Remarks.

All specimens deposited at the National Museum of Natural Science, Taichung were collected using Malaise traps. Many flightless, nocturnal galerucines have been collected in Malaise traps, including *Taiwanoshairachujoi* (Kimoto, 1982) (Lee and Beenen 2020), *Paraplotestaiwana* Chûjô, 1963 (Lee 2015), and *Lochmaealesagei* Kimoto, 1996 (Lee 2019). Moreover, two specimens were collected during the night by Ta-Hsiang Lee (李大翔) and Bo-Xin Guo (郭泊鑫), respectively; they are members of TCRT. These events suggest that adults of *Shairellacaerulea* are nocturnal.

#### Distribution.

This species is probably widespread in Taiwan although few specimens are available for study.

## ﻿Discussion

The former studies have confused the taxonomic boundaries between *Japonitata* and *Paraplotes* (Chen and Jiang 1986; Medvedev 2002; Zhang et al. 2008). This confusion is probably due to overlooking detailed structures of the aedeagus and female genitalic characters. Shapes and structures of the tectum and endophallic sclerites of the aedeagus, and spermatheca in *S.quadricostata* and *S.caerulea* indicate great similarity among both species and species of *Shairella*. Diagnostic characters between *Japonitata*, *Paraplotes*, and *Shairella* are reevaluated and proposed in this study. Transfer of *S.quadricostata* and *S.caerulea* to *Shairella* is supported based on these diagnostic characters. This study also emphasizes the importance of detailed studies and illustrations of male and female genitalic characters.

Presence or absence of hindwings and elytral calli, or shortened elytra are not key characters for generic diagnoses. For example, females of Taiwanese species of *Paraplotes* have reduced hindwings and shortened elytra (Lee 2015). Taiwanese species of *Sikkimia* (Lee and Bezděk 2016) and some species of *Lochmaea* (Lee 2019) have reduced elytral calli and hindwings. The brachelytrous *Shairella* is redefined here by including *S.quadricostata* and *S.caerulea* with normal elytra. This implies that a number of additional *Japonitata* species should be transferred to *Shairealla*. Specifically, species of *Japonitata* without one pair of distinct ridges on the elytra should be evaluated as possible members of *Shairella*.

Adults of *Shairellaquadricostata* (Kimoto), comb. nov. are widespread and some populations have reduced hindwings in mid-elevations of southern Taiwan. They are allopatric with other members of the genus except at Erhwanping (二萬坪) and Hsitou (溪頭), where *S.aeneipennis* Chûjô, 1962 also occurs (Fig. 8). However, they are separated ecologically since both species utilize different food plants (*Hemiboeabicornuta* for *S.quadricostata* and Clinopodiumlaxiflorumvar.taiwanianum for *S.aeneipennis*). Interestingly, adults and larvae of *S.chungi* Lee & Beenen, 2017 in southern Taiwan also feed on leaves of *Hemiboeabicornuta*. This species is allopatric with *S.quadricostata*, although the flightless populations are more northern in distribution and the winged populations are southern. A previous hypothesis for brachelytry in leaf beetles of tropical forest habitats is different from Lee’s proposal for *Paraplotes* (Lee 2015): “Reduction of hind wings may result from the production of physogastric females. Nocturnal behavior increases survival since natural enemies are less of a threat. Males actively search for mates by night. In harsh environments such as islands, deserts and alpine regions, flight is not essential to survival and energy can be diverted to egg production (Beenen and Jolivet 2008). Thus, brachelytry is a predictable evolutionary trend.”. The species (*S.quadricostata*) with long antennae and darker color is adapted to nocturnal activity since natural enemies are less of a threat. Some populations have reduced hindwings as an adaptation to stable microhabitats (mid-altitudes in southern Taiwan). Elytra are reduced further due to allopatric speciation (*S.chungi* Lee & Beenen, 2017). Host plant shifts cause adaptive radiation in these circumstance (*S.aeneipennis*, *S.guoi* Lee & Beenen, 2017, *S.motienensis* Lee & Beenen, 2017, and *S.tsoui* Lee & Beenen, 2017).

## Supplementary Material

XML Treatment for Japonitata

XML Treatment for Japonitata houjayi

XML Treatment for Japonitata jungchani

XML Treatment for Shairella quadricostata

XML Treatment for Shairella caerulea
